# Microbiome Dynamics of a Polychlorobiphenyl (PCB) Historically Contaminated Marine Sediment under Conditions Promoting Reductive Dechlorination

**DOI:** 10.3389/fmicb.2016.01502

**Published:** 2016-09-21

**Authors:** Bruna Matturro, Carla Ubaldi, Simona Rossetti

**Affiliations:** ^1^Water Research Institute – National Research Council, MonterotondoItaly; ^2^ENEA, Technical Unit for Environmental Characterization, Prevention and Remediation, Centro Ricerche Casaccia, RomeItaly

**Keywords:** polychlorobiphenyls, *Dehalococcoides mccartyi*, marine sediments, reductive dechlorination, Epsilonproteobacteria, Dehalococcoidia, next generation sequencing (NGS), microbiome

## Abstract

The toxicity of polychlorinated biphenyls (PCB) can be efficiently reduced in contaminated marine sediments through the reductive dechlorination (RD) process lead by anaerobic organohalide bacteria. Although the process has been extensively investigated on PCB-spiked sediments, the knowledge on the identity and metabolic potential of PCB-dechlorinating microorganisms in real contaminated matrix is still limited. Aim of this study was to explore the composition and the dynamics of the microbial communities of the marine sediment collected from one of the largest Sites of National Interest (SIN) in Italy (Mar Piccolo, Taranto) under conditions promoting the PCBs RD. A long-term microcosm study revealed that autochthonous bacteria were able to sustain the PCB dechlorination at a high extent and the successive addition of an external fermentable organic substrate (lactate) caused the further depletion of the high-chlorinated PCBs (up to 70%). Next Generation Sequencing was used to describe the core microbiome of the marine sediment and to follow the changes caused by the treatments. OTUs affiliated to sulfur-oxidizing ε-*proteobacteria, Sulfurovum*, and *Sulfurimona*s, were predominant in the original sediment and increased up to 60% of total OTUs after lactate addition. Other OTUs detected in the sediment were affiliated to sulfate reducing (δ-*proteobacteria*) and to organohalide respiring bacteria within *Chloroflexi* phylum mainly belonging to *Dehalococcoidia* class. Among others, *Dehalococcoides mccartyi* was enriched during the treatments even though the screening of the specific reductive dehalogenase genes revealed the occurrence of undescribed strains, which deserve further investigations. Overall, this study highlighted the potential of members of *Dehalococcoidia* class in reducing the contamination level of the marine sediment from Mar Piccolo with relevant implications on the selection of sustainable bioremediation strategies to clean-up the site.

## Introduction

Polychlorinated biphenyls (PCBs) form a family of 209 congeners characterized by physical and chemical properties desirable for various industrial and commercial purposes such as dielectric, heat transfer, hydraulic fluids, plasticizers, and fire retardants. Nevertheless, because of their high toxicity, PCBs have been banned since 1970. Nowadays more than 1.5 million tons of PCBs are disseminated into the environment and accumulated in groundwater, soil, and sediments representing a serious risk for ecosystems and human health (Nogales et al., 2011). Despite their persistence into the environment, some microorganisms are able to reduce chlorinated compounds into less toxic or harmless products through the anaerobic reductive dechlorination (RD), a biological redox-based process, which occurs in the presence of an electron donor such as direct H_2_ or fermentable organic substrates (Passatore et al., 2015). The identity and role of microorganisms involved in the RD of chloroorganics have been widely described in several environments (Löﬄer et al., 2013) including contaminated marine sediments (Kormas et al., 2003; Inagaki et al., 2006; Pachiadaki et al., 2010; Pop Ristova et al., 2015). Among known dechlorinating bacteria, *Dehalococcoides mccartyi* (*Chloroflexi* phylum) is considered the most important biomarker of chlorinated ethenes RD (Maymo-Gatell et al., 1997; Löﬄer et al., 2013; Hug and Edwards, 2013; Bedard, 2014).

More recently the RD capability of some strains of *D. mccartyi* (CBDB1, JN, CG3, CG4, CG5) carrying specialized PCB-dechlorinase genes (*pcbA1*, *pcbA4*, *pcbA5*) and other *Chloroflexi* members, similar but distantly related to *D. mccartyi* such as *Dehalobium chlorocoercia DF-1, strain o-17*, phylotype *SF-1* and phylotype VL-CHL1 was shown (Cutter et al., 2001; Bedard, 2008; Zanaroli et al., 2012a; Wang and He, 2013; Wang et al., 2014, 2015; Matturro et al., 2016a,b).

Nevertheless, the knowledge of the biodiversity of PCB dechlorinators is still limited, particularly in contaminated marine sediments where the presence of competing microbial functional groups involved in several biogeochemical cycles (i.e., nitrogen and sulfur cycling) may impact on the RD performances (Cavallo et al., 1999; Zaccone et al., 2005; Maphosa et al., 2012; Sun et al., 2013; Korlević et al., 2015; Quero et al., 2015; Jugder et al., 2016). Several research efforts are nowadays addressed to the exploration of the core microbiome of PCB contaminated marine sediments in order to shed light on the identity of organohalide respiring bacteria known to thrive within mutualistic microbial communities rather than in pure culture.

The present study aimed to investigate the composition and the dynamics of the microbiome of Mar Piccolo (Taranto, Italy), the most polluted coastal areas in Italy characterized by the presence of the largest steel plant in Europe, oil refineries shipbuilding and a list of other anthropic activities that produced a severe environmental contamination since 1960s (Cardellicchio et al., 2007; Franzo et al., 2016). The high levels of heavy metals, polycyclic aromatic hydrocarbons (PAHs), PCBs and dioxins contamination make the Mar Piccolo one of the largest Sites of National Interest (SIN) in Italy for which a remediation strategy is urgently required due to the concentration of hazardous pollutants and to the risk for the human health and the ecology of the surrounding areas.

The study was performed on the marine sediment taken from the most polluted area of Mar Piccolo close to the navy arsenal and the steelworks plant (Sampling station 1l). The microbiome composition was analyzed through a suite of biomolecular tools (Next Generation Sequencing, CARD-FISH and 16SrRNA gene clone library). Furthermore, the dynamics of the main taxa were monitored during a long-term microcosm study conducted under conditions promoting PCB RD (i.e., incubation under strictly anaerobic conditions and addition of a fermentable carbon source). To evaluate the impact of the treatments on the RD process, *D. mccartyi* and reductive dehalogenase genes were quantified and monitored overtime by Real-time PCR (qPCR). The role of microorganisms affiliated to the main retrieved Operational Taxonomic Units (OTUs) belonging to functional groups involved in biogeochemical cycles of marine environments, including *Chloroflexi* members, was also evaluated and discussed.

## Materials and Methods

### Microcosm Set Up

Anaerobic microcosms were set up in duplicate with the marine sediment collected from the Gulf of Taranto (Mar Piccolo, Ionian Sea, Italy – sampling station “1l” 40° 28′ 46 N, 17° 15′ 38 E) (Supplementary Figure S1). The marine sediment was collected just below (~1 cm) the water/sediment interface using polycarbonate sample tubes as described in Franzo et al. (2016).

Sediment samples were immediately transferred to the laboratory for the microcosms set-up. The 90 g of dry weight sediment were anaerobically incubated in sterile 250-mL serum bottles with 70 mL of synthetic marine water as previously described in Matturro et al. (2016b). The bottles were sealed with Teflon-faced butyl rubber stoppers and fluxed with a mixture of N_2_/CO_2_. All microcosms were incubated at 20°C under rotation. After 350 days of anaerobic incubation, lactate was added as fermentable carbon source (0.7 mM). A sterile control microcosm was also prepared with the autoclaved marine sediment and no PCB dechlorination was observed. Chemical and biological analyses were conducted during the anaerobic incubation before (350 days of incubation) and after the lactate addition (420 days of incubation).

### PCB Quantification

Four gams of slurry from each microcosm were collected in 30 mL glass tubes and stored at -20°C until further processing. PCBs were extracted from the slurry collected in each microcosm and then quantified following the procedure reported in Matturro et al. (2016b). Quantification was based on a three-point calibration curve and data were expressed as ng g^-1^ dry sediment.

Data reported in the present study are referred to the PCB quantification performed before and after lactate addition. The detailed composition of the original marine sediment used to set up the microcosm study is reported in Matturro et al. (2016b).

### CARD-FISH

The sediment slurry (1 g dry weight) was anaerobically collected before and after lactate addition from the serum bottles with sterile spatulas under a N_2_ flux. Samples were immediately fixed in formaldehyde (2% vol/vol final concentration) and then processed to extract cells from sediment particles as previously described (Barra Caracciolo et al., 2005), using Optiprep^®^ (Sigma, Italy) as density gradient medium instead of Nicodenz (Sigma, Italy). Extracted cells were filtered through a 0.2 μm polycarbonate membrane (Millipore, 25 mm diameter) by gentle vacuum (<0.2 bar) and used in CARD-FISH assay as previously described (Matturro et al., 2016b). After the hybridization assay, total cells were stained with Vectashield Mounting Medium^®^ with DAPI (Vector Labs, Italy). Cell counting was performed through microscopic analysis on at least 20 randomly selected microscopic fields for each sample. Cell abundances were expressed as cells *per* dry weight of marine sediment (cells g^-1^). Means and standard deviations were calculated with Microsoft Excel^®^.

### DNA Extraction

DNA was extracted from 0.25 g of dry weight sediment in the original sample and from the microcosm before and after lactate addition. The extraction was performed with PowerSoil DNA Isolation kit (MoBio, Italy) within 48 h from each sampling according to the manufacturer’s instructions. DNA was eluted in 100 μL of sterile water and the concentration and purity were determined by NanoDrop 2000c spectrophotometer (Thermo Scientific, USA). Aliquots were stored at -80°C for a few days and then used for Real time PCR quantification (qPCR) and Next Generation Sequencing (NGS).

### Real Time PCR, qPCR

DNA was used for qPCR absolute quantification assays targeting *D. mccartyi* 16S rRNA and reductive dehalogenase genes *tceA, bvcA, vcrA, pcbA1, pcbA4, pcbA5.* qPCR reactions targeting 16S rRNA, *tceA, bvcA, vcrA* genes were performed with TaqMan^®^ chemistry in 20 μL total volume of SsoAdvanced^TM^ Universal Probes Supermix (Biorad, Italy), including 3 μL of DNA as template, 300 nM of each primer and 300 nM of TaqMan^®^ probe composed by 6-carboxyfluoresceine (FAM) as the 5′ end reporter fluorophore and *N,N,N,N*,-tetramethyl-6-carboxyrhodamine (TAMRA) as the 3′ end quencher.

qPCR reactions targeting *pceA, pcbA1, pcbA2, pcbA3* dehalogenase genes were performed with SybrGreen^®^ chemistry in 20 μL total volume of SsoAdvanced^®^ Universal SYBR^®^ Green Supermix (Biorad, Italy) including 3 μL of DNA as template and 300 nM of each primer. Primers and probes used for each reaction are listed in Supplementary Table S1. Standard curves for the absolute quantification were constructed by using the long amplicons method previously reported in Matturro et al. (2013). Each reaction was performed in triplicate with CFX96 Touch^TM^ Real-Time PCR Detection System (Biorad, Italy). Quantitative data were expressed as gene copy numbers g^-1^ sediment, and error bars were calculated with Microsoft Excel^®^ on triplicate reactions for each sample.

### Next Generation Sequencing (NGS)

#### 16S rRNA Amplicon Library Preparation (V1–3)

The procedure for bacterial 16S rRNA amplicon sequencing targeting the V1–3 variable regions is based on Caporaso et al. (2012), using primers adapted from the Human Gut Consortium (Ward et al., 2012). Ten ng of extracted DNA was used as template and the PCR reaction (25 μL) contained dNTPs (400 nM of each), MgSO_4_ (1.5 mM), Platinum^®^ Taq DNA polymerase HF (2 mU), 1X Platinum^®^ High Fidelity buffer (Thermo Fisher Scientific, USA), and barcoded library adaptors (400 nM) containing V1–3 specific primers: 27F AGAGTTTGATCCTGGCTCAG and 534R ATTACCGCGGCTGCTGG. PCR settings: Initial denaturation at 95°C for 2 min, 30 cycles of 95°C for 20 s, 56°C for 30 s, 72°C for 60 s, and final elongation at 72°C for 5 min. All PCR reactions were run in duplicate and pooled afterward. The amplicon libraries were purified using the Agencourt^®^ AMpure XP bead protocol (Beckmann Coulter, USA) with the following exceptions: the sample/bead solution ratio was 5/4, and the purified DNA was eluted in 33 μL nuclease-free water. Library concentration was measured with Quant-iTTM HS DNA Assay (Thermo Fisher Scientific, USA) and quality validated with a Tapestation 2200, using D1K ScreenTapes (Agilent, USA). Based on library concentrations and calculated amplicon sizes, the samples were pooled in equimolar concentrations and diluted to 4 nM.

#### DNA Sequencing

The purified sequencing libraries were pooled in equimolar concentrations and diluted to 4 nM. The samples were paired end sequenced (2 × 301 bp) on a MiSeq (Illumina) using a MiSeq Reagent kit v3, 600 cycles (Illumina) following the standard guidelines for preparing and loading samples on the MiSeq. 10% Phix control library was spiked in to overcome low complexity issue often observed with amplicon samples.

#### 16S rRNA Amplicon Bioinformatic Processing

Forward and reverse reads were trimmed for quality using Trimmomatic v. 0.32 (Bolger et al., 2014) with the settings SLIDINGWINDOW:5:3 and MINLEN:275. The trimmed forward and reverse reads were merged using FLASH v. 1.2.7 (Magoč and Salzberg, 2011) with the settings -m 25 -M 200. The merged reads were dereplicated and formatted for use in the UPARSE workflow (Edgar, 2013). The dereplicated reads were clustered, using the usearch v. 7.0.1090 -cluster_otus command with default settings. OTU abundances were estimated using the usearch v. 7.0.1090 -usearch_global command with -id 0.97. Taxonomy was assigned using the RDP classifier (Wang et al., 2007) as implemented in the parallel_assign_taxonomy_rdp.py script in QIIME (Caporaso et al., 2010), using the MiDAS database v.1.20 (McIlroy et al., 2015). The results were analyzed in R (R Core Team, 2015) through the Rstudio IDE using the ampvis package v.1.9.1 (Albertsen et al., 2015).

Evenness (H) and the taxonomic distinctness (TD) indices were used to describe the biodiversity in the original marine sediment and during the microcosm study and were calculated using Past version 3.10.

### PCR Amplification of 16S rRNA Genes and Cloning

16S rRNA gene of the microbial community in the original marine sediment and in the marine sediment after lactate addition, was amplified using primers 27f (5′-AGAGTTTGATCMTGGCTCAG-3′) and 1492r (5′-TACGGYTACCTTGTTACGACTT-3′) for the Bacteria domain using Hot Start Taq98 (Lucigen, Italy). PCR reactions were performed with the following cycle: 2 min at 98°C, 30 s at 98°C + 30 s at 58°C + 1 min at 72°C for 38 cycles and 15 min at 72°C. PCR products were purified using the QIAquick^®^ PCR purification kit (Qiagen, Milan, Italy). Cloning of PCR products was carried out using pGEM-T Easy Vector System (Promega, Italy) into Escherichia coli JM109 competent cells (Promega, Italy) according to the manufacturer’s instructions. Positive inserts were amplified from recombinant plasmids obtained from white colonies by PCR using the sequencing primers T7f (5′-TAATACGACTCACTATAGGG-3′) and M13r (5′-TCACACAGGAAACAGCTATGAC-3′) and amplicons of 1465 bp length were purified using the QIAquick PCR purification kit (Qiagen, Milan, Italy). 16S rRNA gene sequences of the clone inserts were obtained using the following primers: 530f (5′-GTGCCAGCMGCCGCCG-3′), 926f (5′-AAACTYAAAKGAATTGACGG-3′), 907r (5′-CCGTCAATTCMTTTRAGTTT-3′), 519r (5′-GWATTACCGCGGCKGCTG-3′).

### Phylogenetic Analysis

The 16S rRNA gene sequences were analyzed with the ARB software (Ludwig et al., 2004) using the SILVA 16S rRNA SSU Reference database release 102 (Pruesse et al., 2007). Sequences were analyzed for chimera formation using the chimera-checking tool QIIME Software. The phylogenetic tree was constructed using the maximum likelihood method RAxML (Stamatakis et al., 2008). For the construction of the phylogenetic tree with sequences from the original marine sediment a total of 18 16S rRNA gene sequences were used and *Chlamidyae* was chosen as outgroup (Supplementary Table S3).

For the construction of the phylogenetic tree with sequences from marine sediment after lactate addition a total of 20 rRNA gene sequences were used and *Acidobacteria* was chosen as outgroup (Supplementary Table S4).

### Nucleotide Sequence Accession Numbers

The 16S rRNA gene sequences were deposited in the GenBank database under the PopSet accession number 969532403 (sequences obtained from the original marine sediment) and the PopSet accession number 969532350 (sequences obtained after lactate addition).

## Results

### PCB Reductive Dechlorination

**FIGURE 1:**
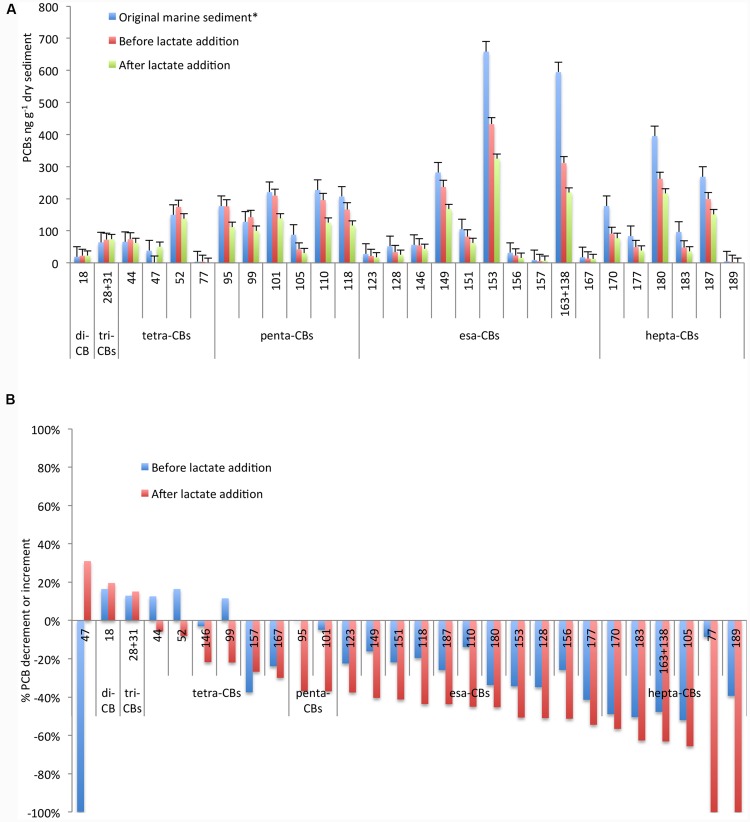
**Concentration of PCB congeners **(A)** and percentage of increment/decrement **(B)** in the original marine sediment and during the microcosm operation before and after lactate addition.** *Data referred to the original marine sediment are from Matturro et al. (2016b).

The sediment was taken from the Station 1l, one of the most polluted areas of the Mar Piccolo heavily contaminated by esa-CBs, penta-CBs, and hepta-CBs (Cardellicchio et al., 2016). In particular, esa-CB 153 and the mixture of PCBs 163+138 were mainly found at concentration >600 ng g^-1^ dry sediment (**Figure 1A**). As demonstrated in a previous treatability study (Matturro et al., 2016b), PCB RD was sustained by the sediment organic carbon under controlled anaerobic conditions with an overall decrement of the most high-chlorinated PCBs up to 50% and the simultaneous increment of low-chlorinated congeners usually found as byproducts of the PCB anaerobic RD (i.e., congeners 18, 28+31) (Bedard, 2008).

In order to further enhance the PCBs dechlorination, in this study we have evaluated the effectiveness of the addition of a fermentable organic substrate. As reported in **Figure 1A**, PCB dechlorination was efficiently promoted and a further decrement of at least 20% up to 70% of the main congeners was observed after lactate addition. Marked decrements were reported for the highest chlorinated congeners 183 (70% of decrement), for the mixture of PCBs 163+138 (63% of decrement) and for the congener 153 (50% of decrement). Additionally, tetra-CB 77 was completely depleted and likely transformed into less chlorinated by-products. In particular, among the screened congeners, tetra-CB 47 strongly increased (from 0.06 to 50 ng g^-1^ dry marine sediment) at the end of the treatment (**Figure 1B**).

### Microbiome Composition and Dynamics by NGS

Next Generation Sequencing analysis of 16S rRNA gene fragments was performed on the original marine sediment, during the anaerobic incubation and after the lactate addition. Sample preparation using the V1–3 bacterial primers were successful for all sampling times and yielded between 16.040 and 28.419 reads after QC and bioinformatic processing. A total of 851 OTUs including 21 OTUs affiliated to unknown phyla and 830 OTUs related to 35 already described phyla was obtained.

The composition of the core microbiome did not drastically change with the incubation under controlled anaerobic conditions. Similar taxonomic distinctness (TD) and evenness (E) indices were estimated on the marine sediment (*TD* = 1.66; *E* = 0.35) and after 350 days of anaerobic incubation (*TD* = 1.72; *E* = 0.39). Conversely, both indices appreciably decreased after lactate addition (*TD* = 1; 1 – *D* = 0.16).

**FIGURE 2:**
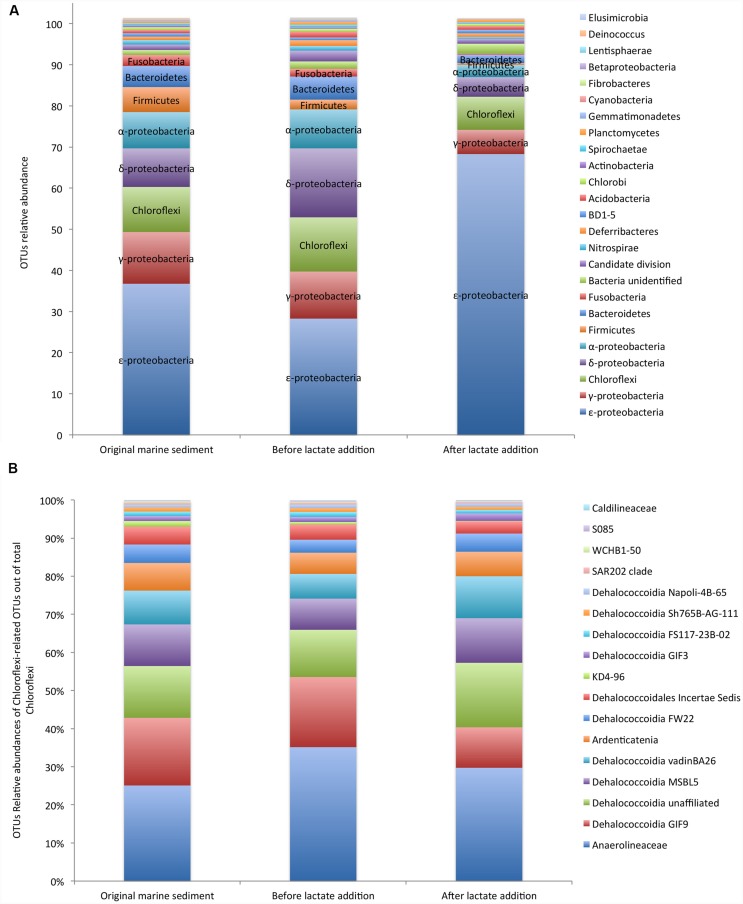
**Core microbiome of the PCB contaminated marine sediment and its evolution after incubation under controlled anaerobic conditions and lactate addition (A).** Relative abundances of *Chloroflexi* members out of total *Chloroflexi* **(B)**. Data are reported as Operational Taxonomic Units (OTUs).

In detail, in the original marine sediment 16S rRNA gene sequences were mainly affiliated to ε-*proteobacteria* (37%), γ-*proteobacteria* (12%), *Chloroflexi* (11%), δ-*proteobacteria* (9%), α-*proteobacteria* (9%), *Firmicutes* (6%) and *Bacteroidetes* (5%) (**Figure 2A**; Supplementary Table S2).

The relative abundances of the OTUs detected in the sediment remained quite constant during the incubation under controlled anaerobic conditions (**Figure 2A**). After the addition of lactate, ε-*proteobacteria* affiliated to *Sulfurovum* and *Sulfurimonas* genera (family *Helicobacteraceae*) increased up to 70% of total OTUs (Supplementary Table S2). *Sulfurovum* was the most abundant genus in the contaminated marine sediment (23.5%) and its relative abundance increased with the treatments reaching the highest value after lactate addition (63.6%). Conversely, *Sulfurimonas* decreased overtime (from 11.7 to 4.68%) (Supplementary Table S2).

OTUs affiliated to γ-*proteobacteria* included iron-oxidizing genus *Acidiferrobacter* and numerous sulfur-oxidizing bacteria such as *Thiomicrospira* genus (*Piscirickettsiaceae)*, *Thioal kalispira* (*Ectothiorhodospiraceae)* and genera *Sedimenticola, Thioalophilus*, and *Marinicella* affiliated to unknown families.

*Chloroflexi* members were mainly affiliated to *Dehalococcoidia* class (≈ 60% of *Chloroflexi*) which includes numerous organohalide respiring microorganisms and, to a lesser extent, to the anaerobic and chemoorganotrophic *Anaerolinea* (≈ 30% of total *Chloroflexi*) and *Ardenticatenia* (<10% of total *Chloroflexi)*, the latter reported to grow by dissimilatory iron- and nitrate-reduction (Kawaichi et al., 2013) (**Figure 2B**). Within *Dehalococcoidia*, *Dehalobium* was the only genus identified by NGS and represented only ≈ 6% of this class in the original marine sediment and <6% after the lactate addition. Among *Chloroflexi*, *Anaerolinea* and *Dehalococcoidia* increased over the anaerobic incubation (Supplementary Table S2).

Members of δ-*proteobacteria* were mainly affiliated to *Desulfobacteraceae, Syntrophobactericeae* and *Desulfoarculaceae* that are strictly anaerobic microorganisms able to use simple organic molecules as electron donors and sulfate or thiosulfate as electron acceptor (Sun et al., 2013). Relative abundance of sequences related to sulfate reducing bacteria increased under controlled anaerobic incubation and declined after lactate addition (Supplementary Table S2). Additionally, OTUs affiliated to α-*proteobacteria* comprised bacteria possessing the biochemical and ecological capacities to degrade organic pollutants and to be resistant to heavy metals such as *Rhodobacterales, Rhodospirillales*, and *Rhizobiales*, the latter recently considered a promising candidate for PCB degradation through oxidative pathways (Teng et al., 2015). In particular, facultative anaerobic photoheterotrophic genera such as *Rhodobium* were among the most abundant OTUs in the contaminated sediment (1.6%) and strongly increased over the anaerobic incubation (up to 4.8%).

*Bacteroidetes* was found at 4.8% in the contaminated sediment and mainly comprised of anaerobic, halophilic bacteria affiliated to *Marinilabiaceae family* and to uncultured *Bacteroidetes* BD2-2.

*Firmicutes* represented 3.3% of total OTUs and were mainly affiliated to *Thermodesulfobiaceae*, which includes several sulfate reducing bacteria (Mori et al., 2003).

Interestingly, evidences of moderately thermophilic and anaerobic chemoorganotrophs capable of fermenting proteinaceous substrates were also found in the marine sediment. In particular, sequences belonging to *Coprothermobacter* spp. (*Firmicutes* phylum) and to *Caldithrix* spp. (*Deferribacteres* phylum) were found and overall accounted for about 3% of total sequences (Supplementary Table S2). Relative abundances of other OTUs were <3% (**Figure 2A**; Supplementary Table S2).

**FIGURE 3:**
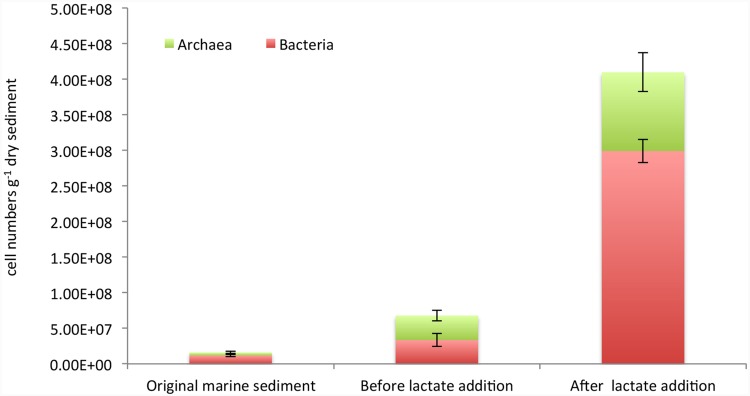
**Cell abundances of total bacteria and archaea estimated by CARD-FISH in the original marine sediment and after the treatments**.

Furthermore, an accurate estimation of *Bacteria* and *Archaea* cell densities was performed by CARD-FISH (**Figure 3**). Bacterial cell abundances accounted for 1.2*E* + 07 ± 1.6*E* + 06 cells g^-1^ dry sediment and increased up to 3.3*E* + 07 ± 9*E* + 06 and 3*E* + 08 ± 1.6*E* + 07 cell numbers g^-1^ dry sediment, respectively, before and after the lactate addition (**Figure 3**). *Archaea* also increased during the treatments from 4.3*E* + 06 ± 1.7*E* + 06 cells g^-1^ dry sediment detected in the original marine sediment to 3.44*E* + 07 ± 7.4*E* + 06 and 1.11*E* + 08 ± 2.7*E* + 07 cells g^-1^ dry sediment quantified in the sediment before and after lactate addition, respectively (**Figure 3**).

### *Dehalococcoides mccartyi* and Reductive Dehalogenase Genes

**FIGURE 4:**
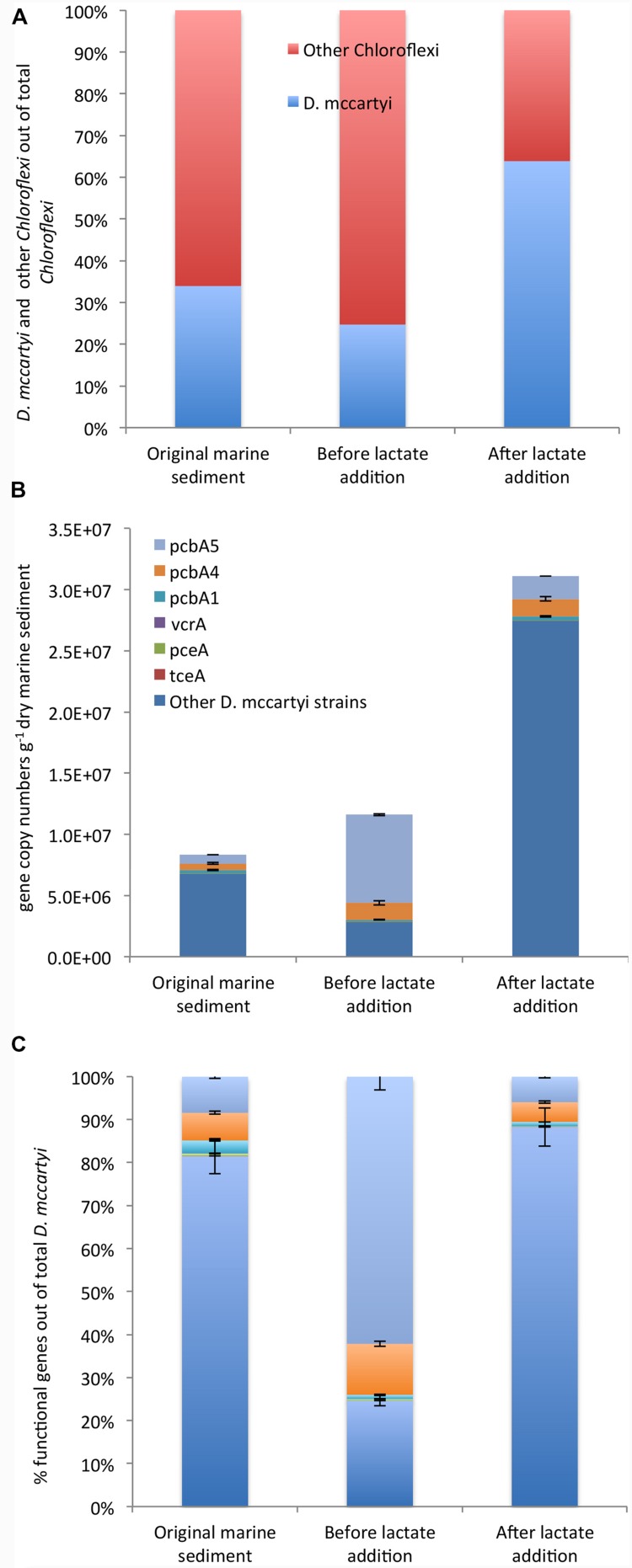
**CARD-FISH quantification of *D. mccartyi* and other members of phylum *Chloroflexi* (A)**. qPCR quantification of *D. mccartyi* strains carrying specific reductive dehalogenase genes and “other” *D. mccartyi* strains not carrying dehalogenase genes here analyzed **(B)**. Relative abundance of each reductive dehalogenase identified out of total *D. mccartyi* 16S rRNA gene copies estimated overtime **(C)**. “Other” *D. mccartyi* strains estimated by subtracting the sum of RDase genes from total *D. mccartyi* 16S rRNA genes.

*Chloroflexi* were quantified by CARD-FISH in the original marine sediment (3.6*E* + 06 cells g^-1^ dry sediment) and increased up to 2.0*E* + 07 cells g^-1^ dry sediment after lactate addition. Among *Chloroflex*i, *D. mccartyi* cells increased overtime representing 25% and 64% of total *Chloroflexi* in the marine sediment and at the end of the treatment, respectively (**Figure 4A**).

Moreover, the occurrence of *D. mccartyi* 16S rRNA and reductive dehalogenase genes (*pceA, tceA, vcrA, bvcA, pcbA1, pcbA4, and pcbA5*) was ascertained and quantified by qPCR (**Figures 4B,C**). A total of 8.33*E* + 06 *D. mccartyi* 16S rRNA gene copies g^-1^ dry sediment were found in the original marine sediment. The anaerobic incubation and the addition of lactate enhanced the total *D. mccartyi* 16S rRNA genes, whose abundances accounted for 1.77*E* + 07 and 3.11*E* + 07 gene copies g^-1^ dry sediment, respectively (**Figure 4B**). *D. mccartyi* strains carrying *pceA*, *tceA*, and *vcrA* genes were found in the original marine sediment at low abundances ranging between 1*E* + 02 – 5*E* + 04 gene copies g^-1^ dry sediment and did not drastically increase during the treatments (**Figure 4B**). Diversely, *D. mccartyi* strains carrying *pcbA5, pcbA4*, *pcbA1* genes were more abundant. Indeed, 7*E* + 05 and 5.4*E* + 05 gene copies g^-1^ dry of *pcbA5* and *pcbA4* genes were detected in the original marine sediment followed by *pcbA1* gene (2.5*E* + 05 gene copies g^-1^ dry sediment). Despite *D. mccartyi* strains carrying PCB-dechlorinase genes increased during the treatments (**Figure 4B**), they represented only a small fraction of total *D. mccartyi* detected by qPCR. Indeed, in the original marine sediment and after lactate addition, *D. mccartyi* strains carrying not identified reductive dehalogenase genes accounted for 80–90% of *D. mccartyi* 16S rRNA gene copies. Diversely, at the end of the anaerobic incubation, where most of the PCB RD was observed, *D. mccartyi* strains carrying *pcbA5* were mainly found and represented 60% of *D. mccartyi* 16S rRNA gene copies (**Figure 4C**).

### Phylogenetic Analysis of Bacterial 16S rRNA Gene Sequences

**FIGURE 5:**
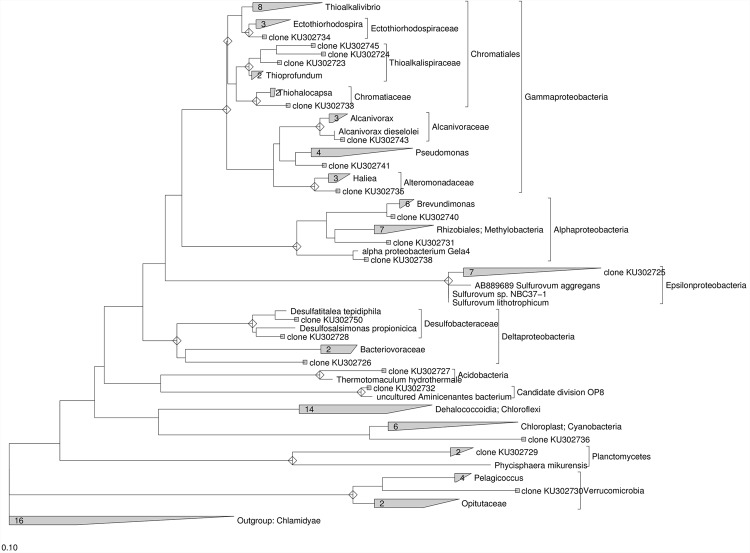
**Maximum Likelihood trees constructed with 16S rRNA gene sequences obtained from the contaminated marine sediment collected at sampling station 1l.** The consensus tree was constructed using the ARB software package. *Chlamidyae* were chosen as outgroups. The scale bar indicates 0.10 changes per nucleotide.

Further insight into the microbial community composition was performed by preparing a 16S rRNA gene clone library with DNA extracted from the marine sediment and at the end of the treatments. The PCR primers used in the clone library allowed the harvest of 16S rDNA from bacterial members in the community. The clone inserts were sequenced and a total of 77 partial or nearly complete 16S rRNA gene sequences were obtained from DNA extracted from the original marine sediment (Supplementary Table S3) and after lactate addition (Supplementary Table S4). Almost all clones obtained from the original marine sediment fell into previously described phyla of the bacterial domain, with the majority being members of the γ-*Proteobacteria* (27%) and ε-*proteobacteria* (27%) phyla. Other clones were in the δ-*Proteobacteria* (11.5%), α-*Proteobacteria* (11.5%), *Planctomycetes* (7.7%), *Verrucomicrobia*, *Acidobacteria*, *Cyanobacteria*, and candidate division OP8 (~4% each) (Supplementary Table S3). Moreover, the phylogenetic analysis of the 16S rRNA gene sequences obtained from the original marine sediment showed that only ≈35% of the clones were closely related to known bacteria with similarities ranging between 97 and 99% (**Figure 5**). They were mainly affiliated to *Sulfurovum aggregans* (ε-*proteobacteria*), *Alcanivorax venustensis* (γ-*proteobacteria*), *Brevundimonas* spp. *Marine-1* (α-*proteobacteria*). Remarkably, most of the clones showed low similarity (≤92%) to known microorganisms and they were phylogenetically located within δ-*proteobacteria, Candidate division OP8, Planctomycetes, Verrucomicrobia.* Several sequences were also found phylogenetically related to *Chromatiales* members (γ-*proteobacteria)* and in particular located within the *Thioalkalispiraceae, Ectothiorhodospiraceae*, and *Chromatiaceae* families. Additionally, the sequence KU302736 was phylogenetically located within the *Chloroplast* lineage and the most close sequences belonged to the *Picea glauca* (white spruce), the latter known to promote microbial biodegradation of PCBs via the release of phytochemicals upon fine root death (Slater et al., 2011).

**FIGURE 6:**
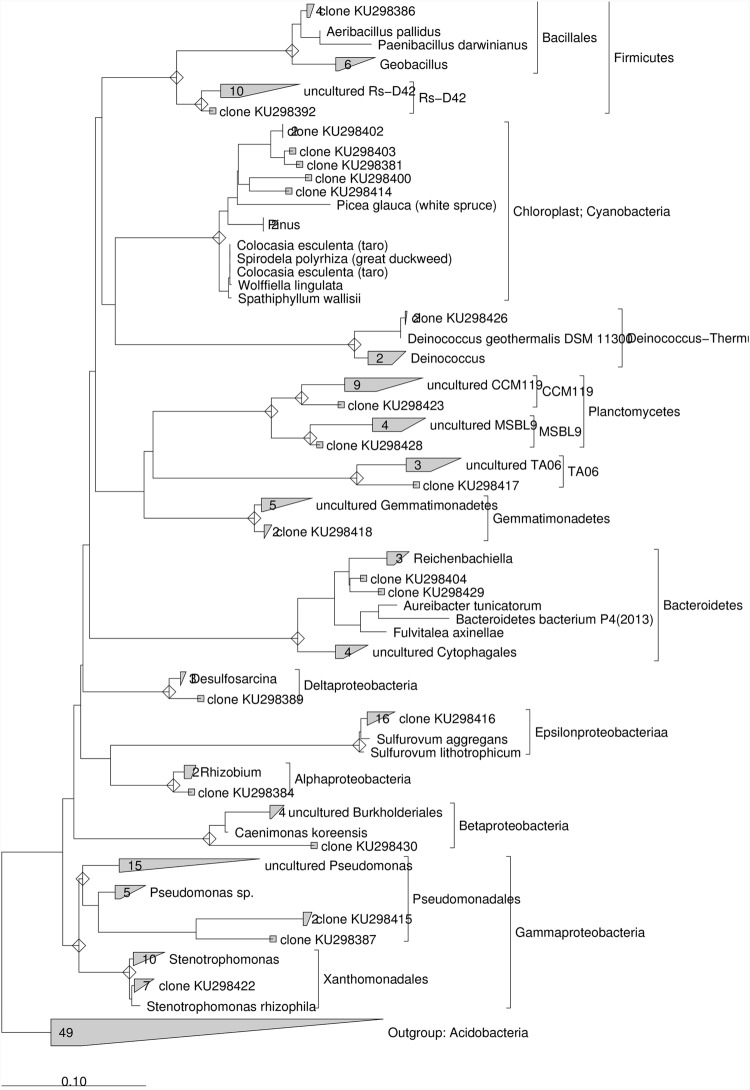
**Maximum Likelihood trees constructed with 16S rRNA gene sequences obtained from the contaminated marine sediment after the lactate addition.** The consensus tree was constructed using the ARB software package. *Acidobacteria* were chosen as outgroups. The scale bar indicates 0.10 changes per nucleotide.

After the addition of lactate, 16S rRNA gene sequences obtained from the contaminated sediment were mostly related to ε-*proteobacteria* (33.3%) and γ-*proteobacteria* (21%). Other clones were in the *Cyanobacteria* (12.5%), *Firmicutes* (8%) and to a lesser extent in the *Deinococcus–Thermus*, *Gemmatimonadetes*, *Planctomycetes*, δ-*proteobacteria*, *Bacteroidetes, Firmicutes, TA06, and Rs-D42* (~ 4% each) (Supplementary Table S4). Phylogenetic analysis conducted on the 16S rRNA gene sequences collected after lactate addition showed that ≈ 76 % of the clones showed similarity ≥97% to known microorganisms (**Figure 6**). Most of these sequences were closely related to *Sulfurovum aggregans* (ε-*proteobacteria*), followed by several sequences phylogenetically related to *Stenotrophomonas rhizophila* (γ-*proteobacteria*), *Rhizobium* (α-*proteobacteria*), *Desulfosarcina* (*δ-proteobacteria*), *Deinococcus geotermalis (Deinococcus-Thermus)* and *Aeribacillus pallidus* (*Firmicutes*). Nevertheless, several sequences showed low similarity to known microorganisms and were phylogenetically related to uncultured *Pseudomonas*, *Caenimonas* members of β-*proteobacteria*, *Bacteroidetes* members close to *Reichenbachiella* lineage, uncultured *Gemmatimonadetes, Planctomycetes* and *TA06.* Sequences phylogenetically related to *Chloroplast* were also retrieved (**Figure 6**).

## Discussion

Polychlorobiphenyls are among the most recalcitrant and toxic compounds to which bioremediation efforts are nowadays addressed. Despite the depletion of these compounds is possible through RD process, only little is known about the identity and role of microbial niches harboring microorganisms adapted at high level of PCB contamination and able to metabolize these compounds, particularly in contaminated marine sediments where complex biogeochemical conditions (i.e., salinity, nitrogen, and sulfur cycling) may strongly affect the RD process.

In the present study, we evaluated the microbial composition and the detoxification potential of the PCB chronically polluted marine sediment collected from the Mar Piccolo of the Gulf of Taranto (Ionian Sea) one of the most polluted coastal areas in Italy. The dynamics of the most abundant taxa and RD biomarkers were monitored during the microcosm study carried out under conditions promoting PCB RD. The process was sustained by the sediment organic carbon, which allowed a decrement of about 50% of the main congeners. This finding is in line with the high organic matter content detected in the marine sediment at sampling station 1l (Total Organic Carbon = 40 mg C g^-1^, Franzo et al., 2016). A further decrease of the contamination level was observed after the addition of a fermentable organic substrate and the main congeners were reduced by at least 20% up to 70%. Overall, the toxicity of PCB contamination decreased with the formation of low chlorinated congeners (e.g., tetra-CB 47, di-CB 18 and tri-CBs 38+31) commonly produced by biological anaerobic RD (Bedard, 2008).

The core microbiome composition of the sediment did not drastically change during the incubation under controlled anaerobic conditions as shown by similar values of the Taxonomic Distinctness and Evenness indices. This is likely due to the anaerobic conditions already existing in the contaminated marine sediment (-400 mV measured already at 0.5 cm below the water/sediment interface, De Vittor et al., 2016) even though the reaction environment is not stable since a resuspension of the superficial sediment caused by boat traffic may occur. Diversely, even though beneficial for the RD process, the addition of a fermentable organic substrate caused a reduction of the microbiome biodiversity and evenness. From an application point of view, the latter finding arises the question whether the need to further refine the sediment bioremediation to achieve the desired outcomes is crucial or not since this may weaken the structure of the microbiota well acclimatized to the contamination. Indeed, the sole incubation under controlled anaerobic conditions has halved the main PCB congeners thus strongly contributing to the reduction of the toxicity of the PCB contamination.

The main components of the microbiome were affiliated to *Proteobacteria* and *Chloroflexi* (representing up to 90% of total OTUs), in line with several previous studies that indicate *Proteobacteria* (particularly δ-*proteobacteria*,γ-*proteobacteria* and ε-*proteobacteria*) as key-bacteria involved in the biodegradation of several organic contaminants associated to sulfur cycling processes in marine sediments (Orcutt et al., 2013). Among these, ε-*proteobacteria* were predominant in the original marine sediment and were further enriched after the lactate addition. In particular, OTUs detected within this subclass were closely related to the genera *Sulfurovum* and *Sulfurimonas*, facultative anaerobes of the *Helicobacteraceae* family that includes members able to survive to high toxic effects and frequently detected at high abundances in marine environments where strong gradients of oxygen and sulfide exist (Inagaki et al., 2004; Campbell et al., 2006; Takai et al., 2006; Nakagawa et al., 2007; Grote et al., 2012; Mino et al., 2014; Mitchell et al., 2014). In particular, *Sulfurovum* is able to grow chemolithoautotrophically with hydrogen, elemental sulfur and thiosulfate as an electron donor and with oxygen, nitrate, thiosulfate, and elemental sulfur as an electron acceptor using CO_2_ as the carbon source. To date only a few complete genomic data are available for this widespread genus, which paves to play a prominent role in the sulfur cycle of contaminated marine sediments (Inagaki et al., 2004; Mino et al., 2014). Additionally, *Sulfurimonas* was also detected in coastal marine sediments and the genomic sequencing of some isolates showed multiple functional genes for different metabolic pathways such as sulfur oxidation, nitrate reduction and hydrogen oxidation, highlighting its metabolic flexibility and similarity to *Sulfurovum* (Sievert et al., 2008).

Interestingly, some evidences suggested the coexistence of these metabolisms with the degradation of chlorinated compounds (Jannasch and Mottl, 1985; Sievert et al., 2008). As the microbial oxidation of reduced sulfur compounds is a key chemolithotrophic process that provides a substantial primary energy source for higher organisms, these observations pose the attention on the needs of deeper investigations on the role of ε-*proteobacteria* in marine sediments contaminated by organic pollutants.

Moreover, OTUs detected within γ-*proteobacteria* subclass were also abundant and were mainly affiliated to *Chromatiales*, anoxygenic phototrophic purple sulfur bacteria, able to perform photosynthesis under anoxic conditions. Among these, species of the subfamily *Chromatiaceae*, generally inhabiting freshwater lakes and intertidal sandflats, and *Ectothiorhodospiraceae*, associated with hypersaline waters, were found. Many of these species oxidize reduced sulfur with and without the aid of anoxygenic photosynthesis. They contain a range of obligate photo- and chemolithotrophs and some organotrophs (Aoyagi et al., 2015) such as *Thioalkalispira* (denitrification-dependent sulfur-oxidizing bacterium*)* and *Acidiferrobacter* (facultative anaerobic iron- and sulfur-oxidizing bacterium). Members of the sulfur-oxidizing family *Piscirickettsiaceae* were also detected, whose presence has been already observed in previous laboratory studies conducted on enrichments obtained from heavily PCB contaminated sediments (Zanaroli et al., 2012b; Koo et al., 2015). Moreover, 16S rRNA gene sequences with low similarity to known γ-*proteobacteria* affiliated to bacteria isolated from contaminated marine environments, such as *Sedimenticola thiotaurini* and overall *Chromatiales* members similar to *Thioprofundum lithotropicum and Thioalkalivibrio thiocyanodenitrificans*, were often found in our samples. Interestingly, recent studies reported the coexistence of these microorganisms with *Sulfurimonas* in marine sediments (Inagaki et al., 2004; Aoyagi et al., 2015). Although, bacteria of the genus *Sulfurimonas* and those belonging to the order of *Chromatiales* employ different metabolic pathways for sulfur oxidation, nitrate reduction and carbon fixation in marine sediments, these sulfur oxidizers were found to coexist and complementary fix carbon, leading to the metabolic activation of fermentative bacteria, ferric ion reducers, and aceticlastic methanogens (Aoyagi et al., 2015). These findings might suggest the occurrence of sulfur oxidation coupled to denitrification during anoxic incubation of the contaminated marine sediment based on chemolithotrophic denitrification-dependent sulfur oxidation. Within γ-*proteobacteria*, sequences homolog to *Stenotrophomonas rhizophila* and *Alcanivorax venustensis*, known for their ability to degrade xenobiotic compounds, were also found by clonal analysis (Wolf et al., 2002).

Within *Firmicutes*, OTUs affiliated to *Coprothermobacter* were found in the original marine sediment even though at a low percentage (2.2%). Interestingly, member of this genus are well known hydrolytic fermentative bacteria and are reported to preferentially use proteins (Gagliano et al., 2014). This is surprising evidence because *Coprothermobacter* spp. are known thermophiles. However, other thermophilic microorganisms were found in the marine sediment as *Caldithrix*, nitrate reducing bacteria retrieved in deep sea hydrothermal vent (Miroshnichenko et al., 2003).

Furthermore, *Chloroflexi* were among the most abundant OTUs detected by NGS and were mainly affiliated to *Dehalococcoidia*, class which comprises many organohalide respiring bacteria (≈ 60% of total *Chloroflexi*) including members of GIF9, MSBL5 and vadinBA26 order and other unaffiliated bacteria (the latter representing about 20% of total *Dehalococcoidia*). They remained quite stable during the microcosm treatments, suggesting their ability to sustain PCB dechlorination in the contaminated marine sediment. Interestingly, no evidence on the occurrence of the phylotype VL-CHL1, a *Chloroflex*i member other than *D. mccartyi* reported for the first time by DGGE as capable of Aroclor1254 dechlorination in marine sediments (Zanaroli et al., 2012a), was found. This finding indicates a widespread dechlorinating capability within *Dehalococcoidia* class and it deserves further research efforts.

*Dehalobium* was the only dechlorinating genus identified by NGS and represented only ≈ 6 % of total *Dehalococcoidia* in the original marine sediment and <6% after the lactate addition.

Nevertheless, CARD-FISH proved that the relative abundance of *D. mccartyi* cells out of total *Chloroflexi* strongly increased after lactate addition suggesting that some OTUs of *Dehalococcoidia* highlighted by NGS might be affiliated to undescribed strains of *D. mccartyi* genus.

Additionally, qPCR quantification of reductive dehalogenase genes proved that *D. mccartyi* strains carrying reductive dehalogenase genes *pcbA5, pcbA4* (i.e., strains CG5 and CG4) and to a lesser extent *pcbA1* (strain CG1) were present in the original marine sediment and increased overtime, demonstrating the occurrence of these *D. mccartyi* strains in PCB contaminated marine sediments where PCB RD occurs. Nevertheless, *D. mccartyi* strains carrying known reductive dehalogenase genes represented a negligible portion of total *D. mccartyi* 16S rRNA genes suggesting that other strains might be likely involved in the PCB RD.

Overall, the outputs of this study highlight the presence and the enrichment of unexplored members of *Dehalococcoidia* in marine sediments where sulfur cycling is predominant and PCB RD processes occur. Recent evidences from single-cell genome sequencing, reported a potential for sulfite reduction as a mode of energy conservation of *Dehalococcoidia* members in marine environments as they may harbor genes encoding dissimilatory sulfite reductase (*dsr* genes) and reductive dehalogenase genes (*rdhA* genes) (Wasmund et al., 2016). This capability in utilizing oxidized sulfur compounds, abundant in marine sediments, as electron acceptors highlights new catabolic potential of *Dehalococcoidia* in marine sediments contaminated by chloroorganics.

## Conclusion

The microbiome analysis of marine sediment collected from one of the most polluted area in Italy (Mar Piccolo, Taranto) revealed the dominance of ε-*proteobacteria* mainly affiliated to sulfur oxidizing bacteria, such as *Sulfurovum*. This group was further enriched in the presence of a fermentable organic carbon (lactate) added to evaluate the effectiveness of this substrate in enhancing the PCB RD through H_2_ production, the actual electron donor of this anaerobic process. The treatment further reduced the main PCB congeners (at least 20–70%) and promoted the growth of specialized dechlorinating bacteria such as *D. mccartyi*. The analysis of the reductive dehalogenase genes known to be involved in the RD of aliphatic and aromatic chloroorganics revealed the presence in the sediment and the enrichment during the treatments of undescribed *D. mccartyi* strains that deserve further investigation. Diversely from the treatment with lactate, the biodiversity of the original sediment resulted mostly unvaried under conditions promoting the PCB RD with H_2_ produced from sediment organic carbon suggesting the capability of the indigenous microbes to efficiently reduce the PCB contamination level of the Mar Piccolo. Overall, this study highlighted the potential of members of *Dehalococcoidia* class in reducing the contamination level of the marine sediment from Mar Piccolo with relevant implications on the selection of proper bioremediation strategies of the site.

## Author Contributions

All authors contributed equally to this work. BM performed the biomolecular experiments, analyzed data, and wrote the paper. CU performed the PCB chemical analysis. SR conceived and coordinated the study and wrote the paper. All authors reviewed the results and approved the final version of the manuscript.

## Conflict of Interest Statement

The authors declare that the research was conducted in the absence of any commercial or financial relationships that could be construed as a potential conflict of interest.
