# Supplementary material for: Microbiome Dynamics of a Polychlorobiphenyl (PCB) Historically Contaminated Marine Sediment under Conditions Promoting Reductive Dechlorination
Source: Front Microbiol. 2016 Sep 21;7:1502. doi: 10.3389/fmicb.2016.01502 (PMC5030254; doi:10.3389/fmicb.2016.01502)
Supplement: Supplementary file 1 [file Data_Sheet_1.PDF]

Supplementary Material

Microbioma dynamics of a polychlorobiphenyl (PCB) chronically contaminated marine sediment under conditions promoting reductive dechlorination

Bruna Matturro, Carla Ubaldi, Simona Rossetti

*** Correspondence:** Corresponding Author: rossetti@irsa.cnr.it

Supplementary Figures and Tables

Supplementary Figure 1. Mar Piccolo, Ionian Sea, sampling station 11 40° 28’ 46 N, 17° 15’ 38 E.

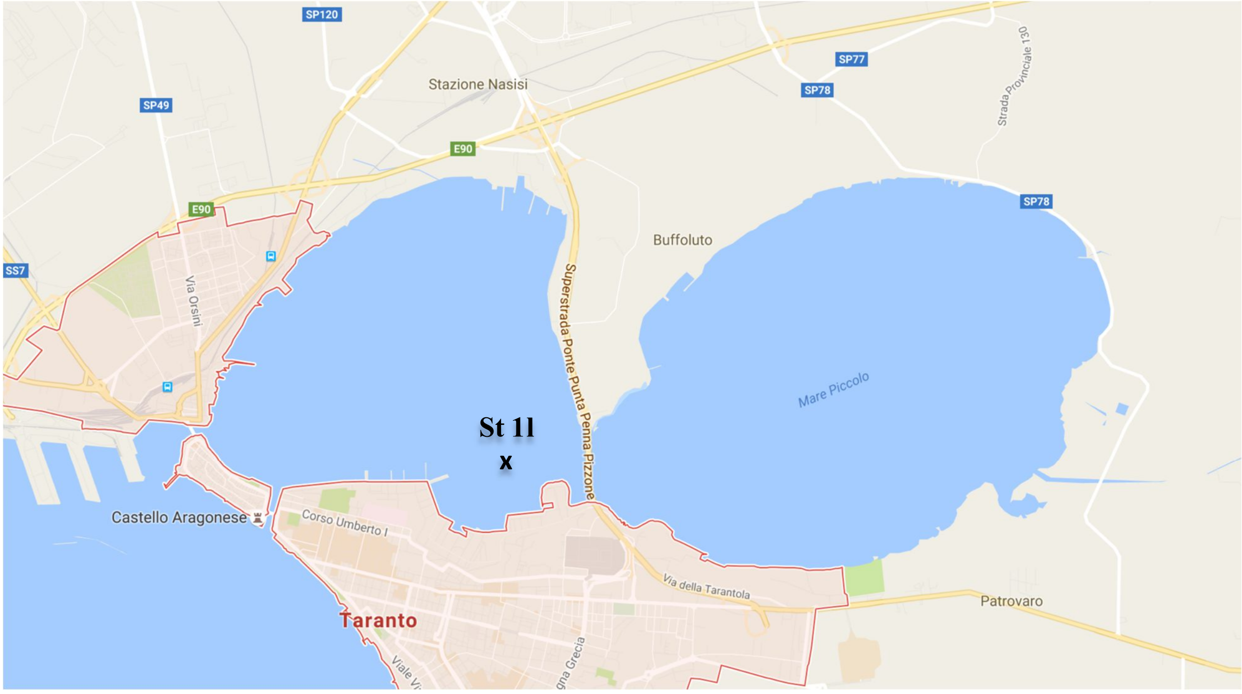

Supplementary Table 1. Primers and probes used for qPCR reactions.

Primers or probe	qPCR chemistry	Target gene	Reference
Dhc 1200F: 5’-CTGGAGCTAATCCCCAAAGCT-3’<br>Dhc 1271R: 5’-CAACTTCATGCAGGCGGG-3’<br>Dhc probe: 5’FAM-TCCTCAGTTCGGATTGCAGGCTGAA-3’TAMRA	TaqMan	16S rRNA	Ritalahti et al., 2006
tceA 1270F: 5’-ATCCAGATTATGACCCTGGTGAA-3’<br>tceA1336R: 5’-GCGGCATATATTAGGGCATCTT-3’<br>tceA probe: 5’FAM-TGGGCTATGGCGACCGCAGG-3’TAMRA	TaqMan	tceA	
bvcA 925F:5’-AAAAGCACTTGGCTATCAAGGAC-3’<br>bvcA 1017R: 5’CCAAAAGCACCACCAGGTC-3’<br>bvcA probe: 5’FAM-TGGTGGCGACGTGGCTATGTGG-3’TAMRA	TaqMan	bvcA	
vcrA 1022F: 5’CGGGCGGATGCACTATTTT-3’<br>vcrA 1093R: 5’GAATAGTCCGTGCCCTTCCTC-3’<br>vcrA probe: 5’FAM-CGCAGTAACTCAACCATTTCCTGGTAGTGG-3’TAMRA	TaqMan	vcrA	
pceA F: 5’-ACCGAAACCAGTTACGAACG-3’<br>pceA R: 5’- GACTATTGTTGCCGGCACTT-3’	SybrGreen	pceA	Beherens et al., 2008
pcbA1 F: 5’-CCGTCAATGGCACTCTGTTCCTTC-3’<br>pcbA1 R:5’-TGCTGGCTTCATTCTCGAAGATCAG-3’	SybrGreen	pcbA1	Wang et al., 2014
pcbA4 F:5’-TGACCAAGGATCTGGTGGAAGGTTG-3’<br>pcbA4 R:5’-AGAAGCGCAATGCCTGAGTGATC-3’	SybrGreen	pcbA4	Wang et al., 2014
pcbA5 F:5’-GGCACAGATGCCTCAAGGAACATAC-3’<br>pcbA5 R:5’-TTGTCCGGCTGCTCCGTCAG-3’	SybrGreen	pcbA5	Wang et al., 2014

Supplementary Table 2. Relative abundances of the microbiome components out of total OTUs detected by NGS in the original marine sediment and in the marine sediment before and after lactate addition. Data are reported as % out of total OTUs.

	Original marine sediment	Before Lactate	After Lactate
Epsilonproteobacteria o__Campylobacterales f__Helicobacteraceae g__Sulfurovum	23.24	22.53	63.55
Epsilonproteobacteria o__Campylobacterales f__Helicobacteraceae g__Sulfurimonas	11.71	5.26	4.68
Gammaproteobacteria o__Chromatiales f__Ectothiorhodospiraceae g__Acidiferrobacter	3.81	3.50	1.36
Chloroflexi c__Anaerolineae o__Anaerolineales f__Anaerolineaceae	2.74	4.63	2.39
Deltaproteobacteria o__Desulfobacterales f__Desulfobacteraceae	2.74	4.48	0.99
Firmicutes c__Clostridia o__Thermoanaerobacterales f__Thermodesulfobiaceae g__Coprothermobacter	2.17	0.09	0.00
Gammaproteobacteria o__Chromatiales f__Ectothiorhodospiraceae g__Thioalkalispira	2.13	1.52	1.64
Chloroflexi c__Dehalococcoidia o__GIF9	1.94	2.43	0.85
Alphaproteobacteria o__Rhizobiales f__Rhodobiaceae g__Rhodobium	1.57	4.75	0.33
Deltaproteobacteria o__Syntrophobacterales f__Syntrophobacteraceae	1.56	3.17	0.42
Chloroflexi c__Dehalococcoidia	1.49	1.63	1.36
Deltaproteobacteria o__Desulfobacterales f__Desulfobacteraceae g__Sva0081 sediment group	1.47	2.67	1.20
Alphaproteobacteria o__Rhodospirillales f__Rhodospirillaceae	1.43	0.90	0.25
Gammaproteobacteria_ unknown	1.25	1.08	0.65
Gammaproteobacteria o__Gammaproteobacteria Incertae Sedis f__Unknown Family g__Thiohalophilus	1.21	0.76	0.71
Fusobacteria c__Fusobacteriia o__Fusobacteriales f__Fusobacteriaceae g__Psychrilyobacter	1.20	0.74	0.16
Chloroflexi c__Dehalococcoidia o__MSBL5	1.20	1.08	0.94
Fusobacteria c__Fusobacteriia o__Fusobacteriales f__Fusobacteriaceae g__Propionigenium	1.10	0.87	0.06
Candidate division OP8	1.10	2.14	1.01
Nitrospirae c__Nitrospira o__Nitrospirales f__Nitrospiraceae	1.08	1.13	0.45
Alphaproteobacteria o__Rhodobacterales f__Rhodobacteraceae	1.08	0.60	0.59
Bacteroidetes c__Bacteroidia o__Bacteroidales f__Marinilabiaceae g__Marinifilum	1.06	0.47	0.16
Epsilonproteobacteria o__Campylobacterales f__Campylobacteraceae g__Arcobacter	1.00	0.17	0.01
Chloroflexi c__Dehalococcoidia o__vadinBA26	0.97	0.85	0.88
Alphaproteobacteria o__Rhizobiales f__Phyllobacteriaceae g__Pseudahrensia	0.94	0.73	0.30
Bacteroidetes c__BD2-2	0.94	1.48	0.74
Gammaproteobacteria o__Order Incertae Sedis f__Family Incertae Sedis g__Marinicella	0.90	1.81	0.27
Alphaproteobacteria o__Rickettsiales f__TK34	0.85	0.75	0.59
BD1-5	0.81	0.65	0.88
Deferribacteres c__Deferribacteres o__Deferribacterales f__Deferribacterales Incertae Sedis g__Caldithrix	0.75	1.26	0.75
Epsilonproteobacteria	0.75	0.26	0.04
TA06	0.74	1.22	0.72
Bacteroidetes c__Cytophagia o__Cytophagales f__Flammeovirgaceae g__Reichenbachiella	0.73	1.05	0.30
Deltaproteobacteria o__Myxococcales f__Sandaracinaceae	0.70	0.75	0.23
Gammaproteobacteria o__Thiotrichales f__Piscirickettsiaceae g__Thiomicrospira	0.62	0.35	0.31
Gammaproteobacteria o__BD3-1	0.62	0.45	0.26
Acidobacteria c__Acidobacteria o__Subgroup 21	0.57	1.07	0.77
Bacteroidetes c__Flavobacteriia o__Flavobacteriales f__Flavobacteriaceae g__Actibacter	0.57	0.80	0.19

Chloroflexi c__Dehalococcoidia o__FW22	0.53	0.45	0.38
Gammaproteobacteria o__Sva0071	0.50	0.48	0.04
Firmicutes c__Clostridia o__Clostridiales f__Clostridiaceae 1 g__Clostridium sensu stricto 17	0.49	0.34	0.10
Deltaproteobacteria o__Desulfarculales f__Desulfarculaceae	0.48	0.69	0.20
Spirochaetae c__Spirochaetes o__Spirochaetales f__Spirochaetaceae g__Spirochaeta	0.47	0.56	0.25
Chloroflexi c__Ardenticatenia	0.45	0.46	0.31
Gammaproteobacteria o__Chromatiales	0.45	0.50	0.28
Alphaproteobacteria	0.45	0.17	0.05
Deltaproteobacteria o__Desulfobacterales f__Desulfobulbaceae g__Desulfobulbus	0.44	0.54	0.11
Bacteroidetes	0.43	0.41	0.06
Deltaproteobacteria o__Sva0485	0.43	0.59	0.20
Actinobacteria c__Acidimicrobiia o__Acidimicrobiales f__OM1 clade	0.42	0.32	0.12
Chlorobi c__Ignavibacteria o__Ignavibacteriales	0.40	0.39	0.33
Alphaproteobacteria o__Rhodospirillales f__Rhodospirillaceae g__Magnetovibrio	0.39	0.11	0.14
Alphaproteobacteria o__Rhizobiales f__Hyphomicrobiaceae g__Filomicrobium	0.37	0.39	0.41
Chloroflexi c__Ardenticatenia o__419 f__2-1	0.35	0.29	0.21
Fusobacteria c__Fusobacteriia o__Fusobacteriales	0.31	0.18	0.00
Proteobacteria c__Deltaproteobacteria o__Myxococcales	0.30	0.29	0.08
Planctomycetes c__Phycisphaerae o__MSBL9	0.30	0.55	0.36
Alphaproteobacteria o__Rhodobacterales f__Rhodobacteraceae g__Pelagicola	0.29	0.12	0.01
Bacteroidetes c__Flavobacteriia o__Flavobacteriales f__Flavobacteriaceae g__Lutimonas	0.27	0.32	0.02
Chloroflexi c__Dehalococcoidia o__Dehalococcoidales f__Dehalococcoidales Incertae Sedis	0.27	0.27	0.15
Alphaproteobacteria o__Rhodobacterales f__Rhodobacteraceae g__Ruegeria	0.27	0.14	0.06
Alphaproteobacteria o__Rhizobiales f__Rhizobiaceae g__Rhizobium	0.27	0.03	0.00
Firmicutes c__Clostridia o__Clostridiales f__Ruminococcaceae	0.26	0.01	0.00
Deltaproteobacteria o__Desulfobacterales f__Desulfobacteraceae g__Desulfatitalea	0.26	2.08	0.63
Gemmatimonadetes c__Gemmatimonadetes o__PAUC43f marine benthic group	0.24	0.37	0.05
Bacteroidetes c__Flavobacteriia o__Flavobacteriales f__Flavobacteriaceae g__Aestuariicola	0.24	0.11	0.00
Gammaproteobacteria o__Gammaproteobacteria Incertae Sedis f__Unknown Family g__Sedimenticola	0.23	0.13	0.10
Firmicutes c__Clostridia o__D8A-2	0.23	0.01	0.00
Bacteroidetes c__Flavobacteriia o__Flavobacteriales f__Flavobacteriaceae g__uncultured	0.22	0.21	0.02
Cyanobacteria c__Chloroplast	0.22	0.23	0.12
Chloroflexi c__Dehalococcoidia o__Dehalococcoidales	0.22	0.23	0.10
Deltaproteobacteria o__Desulfobacterales f__Desulfobulbaceae g__MSBL7	0.22	0.31	0.15
Gammaproteobacteria o__CS-B046	0.21	0.22	0.02
Gammaproteobacteria o__Chromatiales f__Arenicellaceae	0.19	0.29	0.03
Gammaproteobacteria o__E01-9C-26 marine group	0.17	0.13	0.01
Firmicutes c__Bacilli o__Lactobacillales f__Enterococcaceae g__Enterococcus	0.17	0.01	0.00
Deltaproteobacteria o__Desulfuromonadales	0.17	0.39	0.40
Chloroflexi c__KD4-96	0.16	0.09	0.01
Alphaproteobacteria o__Rhodobacterales f__Rhodobacteraceae g__Dinoroseobacter	0.16	0.14	0.01
TM6	0.15	0.16	0.04
Bacteroidetes c__Sphingobacteriia o__Sphingobacteriales f__WCHB1-69	0.14	0.14	0.06

**Chloroflexi c__Dehalococcoidia o__GIF3**	0.14	0.17	0.16
**Gammaproteobacteria o__Xanthomonadales f__JTB255 marine benthic group**	0.14	0.16	0.04
**Chloroflexi c__Dehalococcoidia o__FS117-23B-02**	0.13	0.17	0.07
**Bacteroidetes c__Flavobacteriia o__Flavobacteriales f__Flavobacteriaceae g__Eudoraea**	0.12	0.18	0.06
**Fibrobacteres c__Fibrobacteria o__Fibrobacterales f__09D2Z46**	0.12	0.14	0.09
**Deltaproteobacteria o__Desulfobacterales f__Desulfobacteraceae g__Desulfosarcina**	0.12	0.26	0.13
**Chloroflexi c__Dehalococcoidia o__Sh765B-AG-111**	0.12	0.13	0.07
**Alphaproteobacteria o__OCS116 clade**	0.11	0.10	0.03
**Deltaproteobacteria**	0.11	0.07	0.02
**SHA-109**	0.11	0.10	0.07
**Bacteroidetes c__Flavobacteriia o__Flavobacteriales f__Flavobacteriaceae g__Mesonia**	0.11	0.00	0.00

Supplementary Table 3. 16S rRNA gene sequences of the clone library constructed with DNA extracted from the original marine sediment. Sequences in the same row share 99% of similarity. Nearly complete 16S rRNA gene sequences are marked and were used for the phylogenetic analysis.

**16S rRNA gene sequences obtained from the original marine sediment**	
**Accession number**	**Phylogenetic affiliation**
KU302743*	*γ-Proteobacteria; Alcanivoraceae; Alcanivorax venustensis*
KU302735*	*γ-Proteobacteria; Alteromonadaceae*
KU302733*	*γ-Proteobacteria; Chromatiales; Chromatiaceae; Thiohalocapsa*
KU302723*, KU302724*, KU302745*	*γ-Proteobacteria; Chromatiales; Chromatiaceae; Thioalkalispiraceae*
KU302734*	*γ-Proteobacteria; Chromatiales; Ectothiorhodospiraceae*
KU302738*	*α-Proteobacteria; α-Protoebacterium Gela4*
KU302731*	*α-Proteobacteria; Rhiziobiales*
KU302740*	*α-Proteobacteria; Brevundimonas*
KU302725*, KU302739, KU302748, KU302749, KU302742, KU302744, KU302746	*ε-proteobacteria; Sulfurovum*
KU302726*	*δ-Proteobacteria; Bacteriovoraceae*
KU302750*<br>KU302728	*δ-Proteobacteria; Desulfobacteraceae*
KU302727*	*Acidobacteria*
KU302736*	*Chloroplast; Cyanobacteria*
KU302730*	*Verrucomicrobia*
KU302732*	*Candidate division OP8*
KU302729* KU302737	*Plancomycetes*

Supplementary Table 4. 16S rRNA gene sequences of the clone library constructed with DNA extracted from the marine sediment after lactate biostimulation. Sequences in the same row share 99% of similarity. Nearly complete 16S rRNA gene sequences are marked and were used for the phylogenetic analysis.

**16S rRNA gene sequences obtained after lactate addition**	
**Accession number**	**Phylogenetic affiliation**
KU298422*, KU298412, KU298406, KU298410, KU298378, KU298411, KU298405	*γ-Proteobacteria; Xanthomonadales; Stenotrophomonas rizophila*
KU298415*, KU298425, KU298387*	*γ-Proteobacteria; Pseudomonadales*
KU298430*	β-Proteobacteria; Caenimonas koreensis
KU298416*, KU298406, KU298398, KU298379, KU298391, KU298413, KU298393, KU298380, KU298388, KU298394, KU298385, KU298399, KU298395, KU298421, KU298390, KU298382	*ε-proteobacteria; Sulfurovum*
KU298389*	*δ-Proteobacteria; Desulfosarcina*
KU298429*, KU298404*	*Bacteroidetes*
KU298418*, KU298396	*Gemmatimonadetes*
KU298417*	*TA06*
KU298428*, KU298423	*Planctomycetes*
KU298426*, KU298427	*Deinococcus-Thermus; Deinococcus geothermalis*
KU298381*, KU298400*, KU298414*, KU298403*, KU298402*, KU298383	*Chloroplast, Cyanobacteria*
KU298392*	*Rs-D42*
KU298386*, KU298424*, KU298401, KU298420	*Firmicutes, Bacillales*

References

Ritalahti KM, Amos BK, Sung Y, Wu Q, Koenigsberg SS, Löffler FE. (2006) Quantitative PCR targeting 16S rRNA and reductive dehalogenase genes simultaneously monitors multiple Dehalococcoides strains. *Appl Environ Microbiol.* 72(4):2765-74.

Behrens S, Azizian MF, McMurdie PJ, Sabalowsky A, Dolan ME, Semprini L, Spormann AM. (2008) Monitoring abundance and expression of "Dehalococcoides" species chloroethene-reductive dehalogenases in a tetrachloroethene-dechlorinating flow column. Appl Environ Microbiol. 74(18):5695-703.

Wang S, Chng KR, Wilm A, Zhao S, Yang KL, Nagarajan N, He J. (2014) Genomic characterization of three unique Dehalococcoides that respire on persistent polychlorinated biphenyls. *Proc Natl Acad Sci U S A.* 111(33):12103-8.
